# Observation of aggregation triggered by Resonance Energy Transfer (RET) induced intermolecular pairing force

**DOI:** 10.1038/s41598-017-05157-8

**Published:** 2017-07-20

**Authors:** Xiaoyong Pan, Weizhi Wang, Lin Ke, Nan Zhang

**Affiliations:** 1 2 Fusionopolis Way, Innovis, #08-03, Institute of Materials Research and Engineering (IMRE), 138634 Singapore, Singapore; 2 State Key Laboratory of Molecular Engineering of Polymers, Collaborative Innovation Center of Polymers and Polymer Composite Materials, Department of Macromolecular Science, Fudan University, Shanghai, 200433 China

## Abstract

In this report, we showed the existence of RET induced intermolecular pairing force by comparing their fluorescence behaviors under room illumination *vs* standing in dark area for either PFluAnt solution or PFluAnt&PFOBT mixture. Their prominent emission attenuation under room illumination brought out the critical role of photo, i.e. RET induced intermolecular pairing force in induction of polymer aggregation. Constant UV-Vis absorption and fluorescence spectra in terms of both peak shapes and maximum wavelengths implied no chemical decomposition was involved. Recoverable fluorescence intensity, fluorescence lifetime as well as NMR spectra further exclude photo induced decomposition. The controllable on/off state of RET induced intermolecular pairing force was verified by the masking effect of outside PFluAnt solution which function as filter to block the excitation of inside PFluAnt and thus off the RET induced intermolecular pairing force. Theoretical calculation suggest that magnitude of RET induced intermolecular pairing force is on the same scale as that of van der Waals interaction. Although the absolute magnitude of RET induced intermolecular pairing force was not tunable, its effect can be magnified by intentionally turn it “on”, which was achieved by irradiance with 5 W desk lamp in this report.

## Introduction

Since it was first described over 50 years ago, Förster resonance energy transfer, i.e. fluorescence resonance energy transfer (FRET)^1–4^ has attracted much research attention in both academic and industrial field. As major experimental method to determine the distance between two specific chromophores, FRET relies on the distance dependence of energy transfer from donor molecule to acceptor molecule. FRET has also been effectively used to investigate the intermolecular interaction due to its sensitivity to intermolecular separation, which in turn is affected by their mutual interaction. In most case, FRET was employed as a tool and was described as a process; no attention has been paid to the intermolecular interaction associated with FRET itself^5^. On the other hand, in most text books or journal literatures^6–12^, the introduction and discussion of various intermolecular interactions has been confined to electrostatic interaction, hydrogen bonding, hydrophobic interaction, π-π stacking interaction and Van Der Waals interaction, etc. Other kind of intermolecular interaction has been masked by the above-listed well-known weak interactions and the effort to uncover new intermolecular interaction has been vain.

Here we first unambiguously demonstrated the existence of resonance energy transfer (RET) induced intermolecular pairing force^5^. Two fluorescent polymers, i.e. PFluAnt and PFOBT were employed in this study and the role of photo, i.e. RET induced intermolecular pairing force, which triggered the polymer aggregation under room illumination, was brought out by their dramatic emission decrease (fluorescence attenuation due to aggregation) of solution under room illumination. The controllability, i.e. scalability of this unraveled intermolecular interaction was also investigated.

## Results

**Figure 1:**
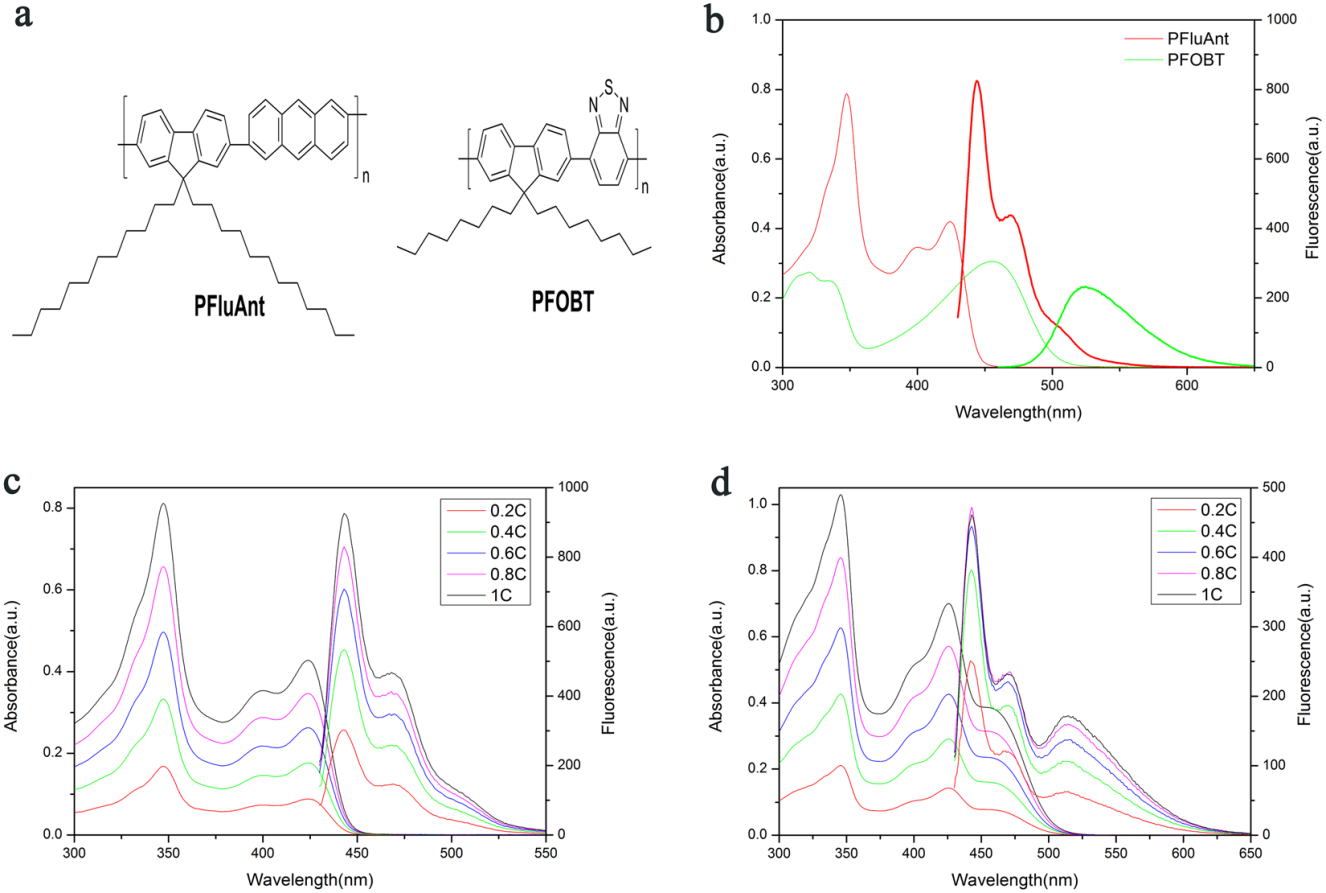
Chemical structures and optical properties of PFluAnt solution and PFluAnt&PFOBT mixture. (**a**) Chemical structures of fluorescent polymers (PFluAnt and PFOBT) used in this study. (**b**) UV-Vis and Fluorescence spectra of PFluAnt and PFOBT solution. (**c**) concentration dependence of UV-Vis absorption and fluorescence spectra of PFluAnt solution. (**d**) concentration dependence of UV-Vis absorption and fluorescence spectra of PFluAnt &PFOBT mixture solution.

The chemical structure as well as UV-Vis absorption and Fluorescence spectra (excited at 424 nm and 455 nm respectively) of PFluAnt and PFOBT in THF were shown in Fig. 1(a and b).

The spectra requirement (overlap between donor emission and acceptor absorption) suggests that PFluAnt and PFOBT will be efficient donor and acceptor for FRET. It is also noteworthy to point out that, due to its low stokes shift^13^, self-absorption of PFluAnt is anticipated to be significant and PFluAnt itself can be both donor and acceptor for FRET. In the following section, unless specified elsewhere, excitation wavelength of mixture solution was set at 424 nm in order to study the FRET because this is the wavelength corresponding to the absorption maximum for donor polymer PFluAnt. Due to the internal conversion, excitation of PFluAnt solution at 347 nm will yield the same emission spectra with lower intensity. It thus can be anticipated that excitation of PFluAnt solution or PFluAnt&PFOBT mixture at 347 nm will exhibit similar effect in terms of aggregation triggered by RET induced intermolecular pairing force while its significance may be suppressed due to donor’s lower emission intensity. As a result, excitation with 347 nm or other wavelength will not be our focus.

The concentration dependence of UV-Vis absorption and fluorescence intensity for both PFluAnt solution and PFluAnt&PFOBT mixture were investigated and the spectra were included in Fig. 1c and d. The concentration *C* stands for 1.2 × 10^−5^ mol/L repeating unit for PFluAnt solution and 1.2 × 10^−5^ mol/L PFluAnt & 9.5 × 10^−6^ mol/L PFOBT repeating units for the mixture solution. It is obvious that the concentration is in the linear range of absorption for both PFluAnt solution and PFluAnt&PFOBT mixture. Nevertheless, the decrease of fluorescence intensity does not follow linearly with dilution of the solution. Due to the suppressed FRET with dilution because of intermolecular distance increase, the decrease of fluorescence intensity lagged much behind that of absorption in the course of dilution.

As fluorescence intensity is more sensitive to external environmental change^13^, we track their fluorescence intensity after different standing time, either under room illumination or standing in dark area. Another reason why we paid more attention to fluorescence intensity change is that we employ FRET as analytical tool to monitor the distance change between donor and acceptor due to the strong distance dependence of resonance energy transfer efficiency.

**Figure 2:**
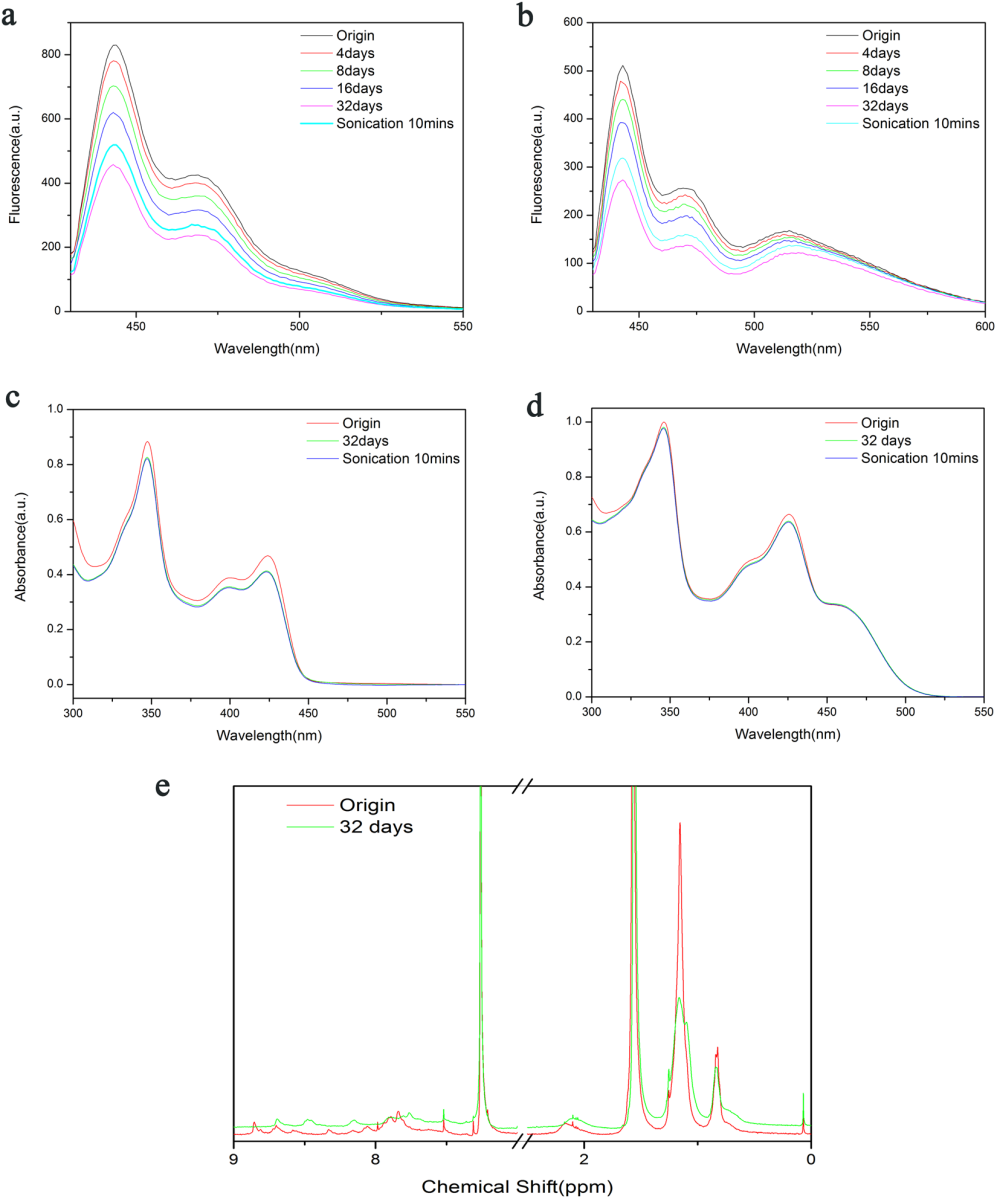
Optical spectra of PFluAnt solution and mixture solution as well as and NMR spectra of PFluAnt solution. (**a**) Fluorescence spectra of PFluAnt solution under room illumination after different standing time. (**b**) Fluorescence spectra of PFluAnt&PFOBT mixture under room illumination after different standing time. (**c**) UV-Vis absoption spectra of PFluAnt solution under room illumination after 32 days’ standing time. (**d**) UV-Vis absoption spectra of PFluAnt&PFOBT mixture under room illumination after 32 days’ standing time. (**e**) NMR spectra of original PFluAnt solution in CDCl_3_ and that after 32 days’ room illumination.

From Fig. 2a and b, we noticed much suppressed emission (at ~443 nm) from mixture as contrast to PFluAnt solution although the concentration of PFluAnt were kept the same and the emission at 443 nm comes solely from PFluAnt. The decrease of mixture emission at ~443 nm with respect of that of PFluAnt solution is due to the energy transfer between PFluAnt and PFOBT as well as the “filter” effect^13^ of PFOBT on PFluAnt absorption. As contrast to the constant fluorescence intensities (Fig. S1a and b) of both PFluAnt and PFluAnt&PFOBT mixture standing in dark area, the decrease of their fluorescence intensities under room illumination is dramatic, which cannot be explained by systematic fluctuation. As a contrast, the fluorescence intensity of PFOBT is quite stable (Fig. S1c) no matter it is under room illumination or standing in dark area. In the case of the mixture, observable bathochromic shift of long wavelength peak (~515 nm) can be ascribed to the increased emission from PFOBT with respect to that of PFluAnt. This is explainable in the context of increased energy transfer from PFluAnt to PFOBT associated with their decreased distance, *i.e. polymer aggregation* due to RET induced intermolecular pairing force (all other possibilities, *i.e. concentration variation, polymer decomposition etc*. which may result in the decrease of fluorescence intensity have been excluded in the following sections). The decrease of the absolute value of the peak intensity at ~515 nm was resulted from the decrease of the long wavelength emission from donor polymer PFluAnt, which overwhelmed the increase of ~515 nm emission from acceptor polymer PFOBT. (No normalization was conducted to compare the relative emission from PFOBT and that from PFluAnt as this may result in the increase of intrinsic emission contribution from PFOBT with longer standing time.)

The fluorescence intensity is partially recoverable by sonication in water bath for only 10 minutes, which suggests that the decrease of fluorescence intensity is unlikely due to the photo induced decomposition.

UV-Vis absorption spectra of original solution as well as solution after 32 days’ room illumination have been collected for PFluAnt solution as well as PFluAnt&PFOBT mixture and were also shown in Fig. 2(c and d). It is obvious from Fig. 2c and d that there is no change of either peak shape or peak wavelength for their UV-Vis absorption. The observation is the same for PFluAnt emission while the slightly different variation of PFluAnt&PFOBT mixture emission is explainable in the context of increased FRET and suppressed filter effect. However, the intensity of absorption maximum exhibit certain decrease for both PFluAnt solution and PFluAnt&PFOBT mixture after 32 days’ room illumination. This implied the formation of strong aggregation, which was strengthened further by Van Der Waals interaction and π-π stacking interaction^14–16^. The somewhat suppressed filter effect (from Fig. 2d) of PFluAnt on PFOBT may also contribute to the fluorescence bathochromic shift in Fig. 2b. The suppressed absorption at 424 nm, which is more likely from PFluAnt absorption, increased the exposure of PFOBT to light source so that its emission was increased. Although π-π stacking interaction is not effective to trigger aggregation, it may be strong once it is slowly accumulated. This is why restoration of UV-Vis absorption cannot be observed after sonication for 10 minutes for both PFluAnt solution and PFluAnt&PFOBT mixture (Fig. 2c and d).

The constant shapes of both UV-Vis absorbance and fluorescence spectra suggest that photo induced polymer decomposition is unlikely. Solvent molecules (in the vicinity of polymer) reorientation may also be excluded since this may cause the shift of absorption & fluorescence wavelengths^13, 17^. Moreover, the 32 days’ standing time is too long for solvent molecules reorientation.

For both PFluAnt solution and PFluAnt&PFOBT mixture, the much faster decrease of emission intensities as contrast to that of absorption intensities implies that photo-induced solubility decrease is unlikely since (From Fig. 1c and d) dilution of solution will result in lagged fluorescence intensity decrease.

Nevertheless, the absorbance intensities are quite stable for those solutions standing in dark area (Fig. S2a and b). With similarity to fluorescence attenuation, their different absorbance behavior (room illumination *vs* dark standing) implied the existence of RET induced intermolecular pairing force. The argument is corroborated by the stable fluorescence intensity of PFOBT (Fig. S1c) under room illumination due to the absence of resonant energy transfer. It’s more interesting to note that the decrease of absorption peak intensity is more obvious for PFluAnt than PFluAnt&PFOBT mixture. This may be due to the facilitated π-π stacking interaction in PFluAnt as contrast to PFluAnt&PFOBT mixture. It is imaginable that the higher structure similarity may accelerate π-π stacking^14, 18–22^ in PFluAnt while structure incompatibility may, to some extent, suppress the stacking in PFluAnt&PFOBT mixture.

The above observation suggested that with respect to Van Der Waals interaction and π-π stacking interaction, which both existed when the solutions stand in dark area, the magnitude of RET induced intermolecular pairing force is not trivial as the combination of Van Der Waals interaction and π-π stacking interaction cannot induce aggregation even after 32 days’ standing while RET induced intermolecular pairing force can trigger observable aggregation in much shorter time. However, Van Der Waals interaction and π-π stacking interaction cannot be excluded in the intermolecular force responsible for the polymer aggregation during room illumination. Nevertheless, RET induced intermolecular pairing force will trigger aggregation and facilitate π-π stacking while Van Der Waals interaction and π-π stacking interaction strength the aggregation. The aggregation may be strong enough to induce the change of UV-Vis absorption once it is slowly formed. This is why restoration of UV-Vis absorption cannot be observed after sonication for 10 minutes for both PFluAnt solution and PFluAnt&PFOBT mixture. As a contrast, the partial recovery of fluorescence for the same solutions implied that there is additional kind of intermolecular arrangement which can be broken by sonication that is responsible for fluorescence decrease. This additional kind of “looser” intermolecular arrangement can be ascribed to the intermolecular separation without either ordered orientation arrangement or short enough distance to induce observable change of UV-Vis absorption, which is understandable since fluorescence is more sensitive to environmental change than UV-Vis absorption.

NMR spectra of the freshly prepared PFluAnt solution as well as that after 32 days’s room illumination (Fig. 2e) confirmed the prominent π-π stacking^23, 24^ after 32 days’ room illumination. Freshly prepared PFluAnt solution showed the resonance peaks of anthracene moiety protons at 8.9, 8.7 and 8.6 ppm while the signal of fluorene ring proton appeared from 7.7 ppm to 8.0 ppm. The resonance of dodecyl proton arose between 2.2 ppm and 0.8 ppm. As rapid rotational Brownian diffusion of molecules in liquid samples averages out dipolar and other anisotropic magnetic interactions local to NMR-active nuclei in the molecule^23^, the obviously broaden (increased line-width at half height) signal from alkyl side chain (~1.15 ppm and 0.83 ppm) is one of the intrinsic characteristic associated with π-π stacking due to reduced motional narrowing of their signal. The broaden effect is relatively weaker for protons at ~0.83 ppm (as contrast to those at ~1.15 ppm) because they are located farther from the π-π stacked aromatic polymer backbone. Their motional narrowing effect is thus less influenced. Observable up-field shift of signal from protons (~2.2 ppm) in vicinity of aromatic backbone is due to the interaction of these protons with the π-electron field of the π-π stacked aromatic polymer backbone^23^. The shielding effect may be even stronger for aromatic backbone protons due to π-π self-stacking interaction of polymer main chain in the CDCl_3_ solution^23^. This accounts for the obvious up-field shift of aromatic proton signal after 32 days’ room illumination. However, due to different shielding effects which experienced by different aromatic protons, their shifts as well as broaden effects may be different^23^. This is why, after 32 days’ room illumination, the signal from aromatic protons did not exhibit proportionality to that of the freshly prepared solution in terms of either chemical shift or signal intensity. The role of RET induced intermolecular pairing force in triggering aggregation and facilitating π-π stacking interaction is manifest as no observable NMR difference (Fig. S3a) can be observed for PFluAnt solution standing in dark area. The stable NMR spectrum of PFOBT (Fig. S3b) corroborated the role of RET induced intermolecular pairing force.

**Figure 3:**
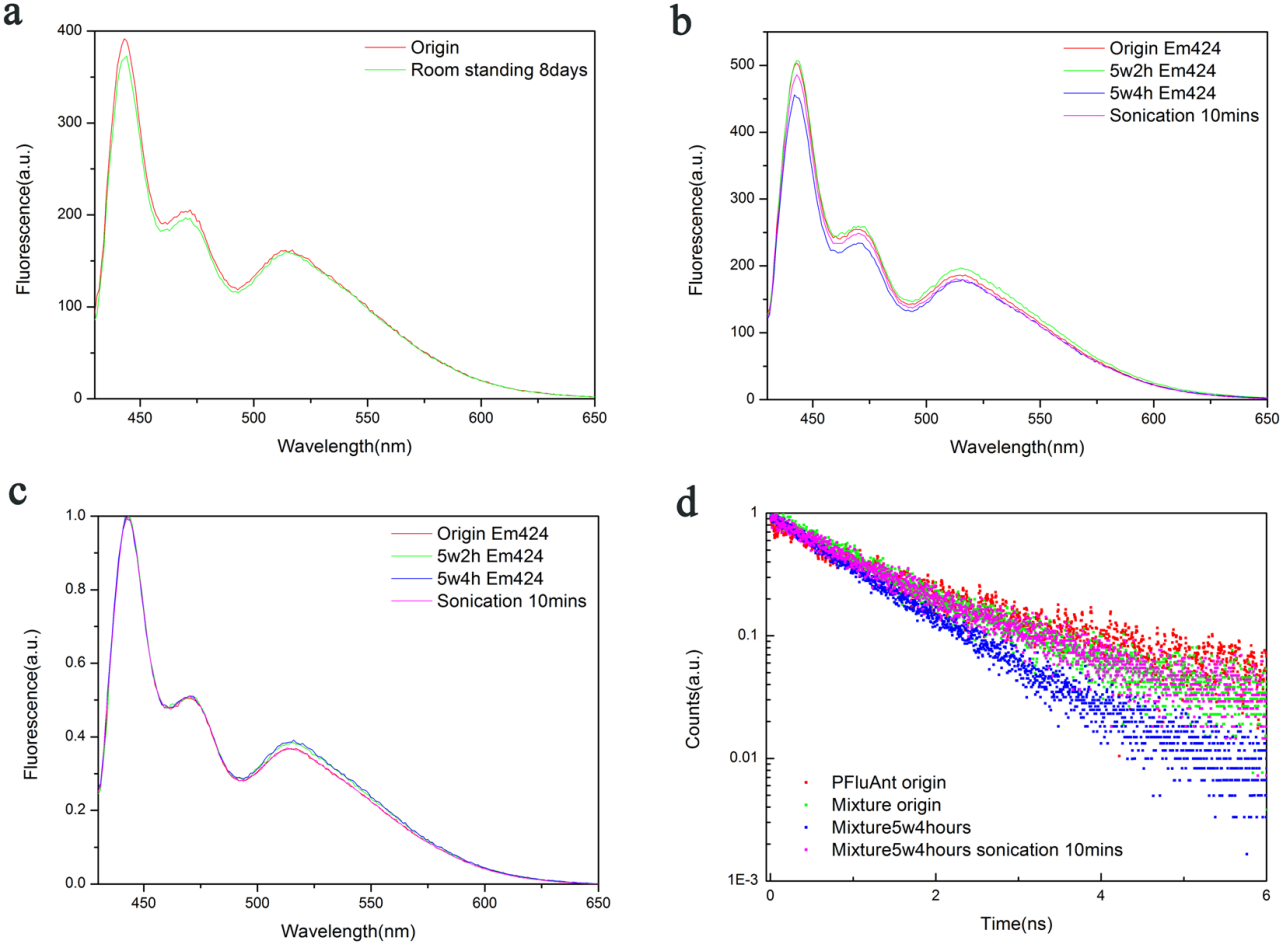
Steady state and time-resolved fluorescence spectra of PFluAnt solution or PFluAnt&PFOBT mixture. (**a**) Fluorescence spectra of PFluAnt&PFOBT mixture in PFluAnt solution after 8 days’ standing under room illumination. (**b**) Fluorescence spectra of PFluAnt&PFOBT mixture after irradiance with 5 W desk lamp. (**c**) normalized spectra of PFluAnt&PFOBT mixture after irradiance with 5 W desk lamp. (**d**) Time resolved fluorescence spectra of PFluAnt solution and PFluAnt&PFOBT mixture.

In order to verify the critical role of excitation of PFluAnt in the mixture aggregation, we intentionally put the mixture in sample cuvette in PFluAnt solution (with its concentration four times that of PFluAnt inside the mixture solution) and let the solution stand for 8 days under room illumination before conducting fluorescence characterization and the spectra were shown in Fig. 3a. Mixture solution was employed in this study due to the more efficient energy transfer from PFluAnt to PFOBT as contrast to that among PFluAnt itself. (The shortest effective light path of the filter solution is only ~3 mm.) The result indicated that the mixture fluorescence intensity decrease was much suppressed as compared to that of mixture solution without PFluAnt mask (Fig. 2b). This further verified our argument as the mask of the mixture solution with PFluAnt will substantially block the excitation of donor polymer PFluAnt. With the donor excitation suppressed, FRET is attenuated and the associated FRET induced intermolecular pairing force is partially tuned off. As a result, the polymer aggregation is not as obvious as that of mixture solution without PFluAnt mask.

In order to investigate the tunability as well as recoverability of the aggregation triggered by RET induced intermolecular pairing force, we irradiated the mixture with 5 W desk lamp before UV-Vis absorption, fluorescence as well as time resolved fluorescence characterization. Again, PFluAnt&PFOBT mixture rather than PFluAnt solution was used in this investigation in order to suppress the π-π stacking interaction. The fluorescence spectra of the original solution, solution after 5 W illumination as well as solution after subsequent sonication for 10 minutes was show in Fig. 3b.

(Fluorescence spectra of PFluAnt&PFOBT mixture after 4 hours’ standing under room illumination, UV-Vis absorption spectrum of PFluAnt&PFOBT mixture after irradiance with 5 W desk lamp for 4 hours were also collected and were shown in Fig. S4).

The decrease of PFluAnt fluorescence intensity at 443 nm indicated that the aggregation is much more obvious for solution irradiated with 5 W desk lamp as contrast to that under room illumination even though their standing times are the same. Even though more focused area as well as shorter distance between desk lamp and mixture solution ensured its somewhat higher irradiance, 5 W power supply may not be too much stronger than room illumination. This indicated that the effect of RET induced intermolecular pairing force can be conveniently magnified by intentionally turn it “on” via stronger continuous irradiance. To eliminate the effect of fluorescence intensity fluctuation, we normalized the spectra (Fig. 3b) at 443 nm and the normalized spectra were shown in Fig. 3c. The normalized spectra indicate increased PFOBT emission with respect to PFluAnt emission after 5 W irradiance. Since the absorption fluctuation is neglectable (Fig. S4b), the increase of PFOBT emission implied the increased resonance energy transfer due to aggregation triggered by RET induced intermolecular pairing force. Moreover, the steady state fluorescence is almost completely recoverable (from Fig. 3b and c) which is due to the suppressed π-π stacking interaction with the mixture solution as well as short standing time, which further attenuate the accumulation of π-π stacking interaction.

Time resolved fluorescence spectra shown in Fig. 3d verified the energy transfer from PFluAnt to PFOBT as the lifetime of PFluAnt decreased from 1.62 ns to 1.35 ns. Illumination with 5 W desk lamp for 4 hours further decrease its lifetime to 1.05 ns, which verified the increased energy transfer due to distance decrease. Its lifetime, i.e. 1.39 ns, was completely recovered after 10 mins sonication. It’s noteworthy to point out that all solutions showed a single exponential fit except the difference in their lifetimes which indicates that they all have single excited specie. This is not surprising as emission at 443 nm was monitored, which means only PFluAnt emission was tracked. While the decrease of the distance between PFluAnt and PFOBT facilitated the non-radiative decay and shorten the lifetime of PFluAnt, no new species was generated. Further analysis suggests that, even though the intermolecular distance may be altered upon illumination with 5 W desk lamp, the change is homogeneous for all species, *i.e. donor-acceptor pair*, inside the solution. This implies that aggregation triggered by RET induced intermolecular pairing force with 5 W desk lamp is somewhat slow process on the time scale of hours so that polymeric conformation relaxation/adjustment is feasible, which prevent the observation of micro-scale heterogeneous aggregation.

### Theoretical calculation

RET induced intermolecular pairing force has been observed in somewhat more complicated system of conjugated polymer and carbon nanotubes^5^. Although the observation is not so conclusive, we have derived one equation to calculate the magnitude of RET induced intermolecular pairing force^5^. In order to estimate the magnitude and evaluate its importance with respect to other kind of weak interaction, here we compare the relative scales of Van Der Vaals interaction as well as RET induced intermolecular pairing force.

The intermolecular interaction energies between two free rotating molecules in their ground states can be expressed by the following equation, which is the van der Waals-Londen dispersion energy^25, 26^.

1 $${\rm{\Delta }}ENZ=-\frac{3}{64{\pi }^{2}{\varepsilon }_{0}^{2}{R}^{6}}\alpha (A;0)\alpha (B;0)\hat{E}$$

Where α(ε;0) (ε = A, B) is the static isotropic electric dipole polarizability of molecules ε; ε_0_ and *R* stand for vacuum permittivity and intermolecular separation respectively while the scaled energy is

2 $$\hat{E}=\frac{2{E}^{A}{E}^{B}}{{E}^{A}+{E}^{B}}$$

With E^ε^(ε = A, B) denoting the lowest-energy transition in molecules ε.

The expression for the RET-induced intermolecular pairing force with molecular and pair orientational averaging is listed below^5^:

3 $$ < {\rm{\Delta }}ENZ > =-\frac{11\hslash c{\kappa }^{11/2}{\kappa ^{\prime} }^{1/2}}{960{\pi }^{4}{\varepsilon }_{0}^{2}R}\alpha (D;\kappa )\alpha (A;\kappa ^{\prime} )$$

Where *k* and *k*’ represent the wave vectors corresponding to the emission wavelength of the donor and absorption wavelength of acceptor chromophores and α(D,*k*) and α(A,*k*’) are the molecular dynamic polarizabilities of donor and acceptor molecules at their correspondent wave vectors. *ℏ* and *ϲ* stand for the reduced Planck constant and speed of light transmission in vacuum while other parameters are of the same physical meaning as those in the equation (1).

Theoretical calculation (with details outlined in supporting information) implies that, with 1/20^27, 28^ as approximate value of α(A;0)/α(A;*κ*) where *κ* stands for wave vector at resonant frequency,

4 $${\rm{\Delta }}ENZ/ < {\rm{\Delta }}ENZ > =\frac{497.4}{400}=1.24$$

This suggests that the magnitude of van der Waals interaction may be somewhat higher than RET-induced intermolecular pairing force although they are comparable.

Nevertheless, the significance of RET-induced intermolecular pairing force is anticipated to be higher since the intermolecular separation *R* should be greater than our estimation based on repeating unit concentration rather than polymer chain concentration.

The equation governing the magnitude of RET induced intermolecular pairing force suggests the inverse dependence on intermolecular distance R, indicating that it is a long-range interaction as compared to Van Der Waals interaction, which is a short range interaction with inverse dependence on R^6^. Besides molecular dynamic polarizabilities, the equation also implies the critical role of wavelength at which RET happens, with RET induced intermolecular pairing force approximately scales with λ^−6^ where λ is the resonance wavelength.

## Conclusion

The evidence presented here all are consistent with the existence of RET induced intermolecular pairing force with its magnitude expressed by equation (3). The UV-Vis absorption and fluorescence spectra exhibit stable shapes and peak wavelengths, which implies photo decomposition is unlikely. More importantly, NMR spectra verified strong π-π stacking interaction rather than chemical decomposition. The recoverability of fluorescence, either steady state fluorescence intensity or PFluAnt lifetime in the presence of PFOBT further exclude photo decomposition. The controllable on/off state implied by contrasting behavior under room illumination *vs* that standing in dark area and the masking effect by PFluAnt solution as well as its scalable effect suggested by the much faster aggregation triggered by irradiance with 5 W desk lamp all confirmed the existence of RET induced intermolecular pairing force with non-trivial scale. This newly identified weak interaction may resolve many puzzling observations for fluorescent materials. The feasibility to magnify its effect by stronger continuous irradiance suggests its great potential in various researches and applications.

## Materials and Methods

### Materials

Anhydrous THF were supplied by sigma-aldrich and were stabilized by butylated hydroxytoluene. Both polymers PFluAnt (M_n_ 18700) and PFOBT (M_n_ 8000) were synthesized and the synthetic work will be reported elsewhere. Approximate 16.25 mg/L (2.4 × 10^−5^ mol/L repeating unit) of PFluAnt and 10 mg/L (1.9 × 10^−5^ mol/L repeating unit) of PFOBT in THF were prepared and kept as stock solutions. Equal volume of PFluAnt and PFOBT solution were combined to prepare the PFluAnt&PFOBT mixture solution for further test while the same volume of PFluAnt solution and THF were mixed to get PFluAnt solution for tracking. During the whole tracking period, solutions under room illumination were put in the same place on the same bench. Besides ceiling light, external illumination source was eliminated so that the illumination intensity is comparable. Solutions “standing in dark area” were kept in cabinet and covered with aluminum foil. The stock solutions from which the spectra in the same figure were collected were kept the same although slight variation may exist for stock solutions from which spectra in different figures were collected.

### Methods

^1^H NMR spectra were recorded on a Bruker ACF 400 FT-NMR spectrometer operating at 400 MHz. Deuterated chloroform was used as the solvent, and tetramethylsilane (TMS) was used as the internal standard. The UV-Vis absorption and fluorescence measurements of polymer solutions were conducted on a Shimadzu UV-1601 PC UV-Vis spectrophotometer and a Perkin-Elmer Instrument LS 55 luminescence spectrometer, respectively. The fluorescence lifetime was measured using an IBH FlouroCube time-correlated picosecond single photon counting system (TCSPC). Solutions were excited with a pulsed diode laser (404 nm, < 100 ps pulse duration) with a repetition rate of 1 MHz and the emission at 443 nm was monitored. The fluorescence lifetime values were determined by fitting the data with exponential decay using *origin* software.

## Theoretical Calculation

We estimate the intermolecular separation *R* before we compare the relative scales of Van Der Waals interaction and RET induced intermolecular pairing force because both of them depend on the value of *R*. The concentrations of repeating units of PFluAnt and PFOBT in THF are 2.4 × 10^−5^ mol/L and 1.9 × 10^−5^ mol/L respectively. We ignore the difference of donor and acceptor in order to simplify the estimation, the overall concentrations of both donor and acceptor repeating units is then 2.15 × 10^−5^ mol/L. The volume occupied by each repeating unit is thus 10^24^/(2.15 × 10^−5^ × 6.02 × 10^23^) = 7.726 × 10^4^ nm^3^, which suggests that the intermolecular separation to be 42.6 nm if we treat donor and acceptor molecules as objects with infinite small dimension and assume that each donor and acceptor locates in the middle of each volume it occupied. The wave vectors corresponding to the absorption wavelengths of the donor and acceptor chromophores were both taken as 2π/(443 nm) = 1.42 × 10^7^ m^−1^ in order to further simplify the calculation.

The ratio of van der Waals interaction to RET-induced intermolecular pairing force can thus be expressed as:

5 $${\rm{\Delta }}ENZ/\langle {\rm{\Delta }}ENZ\rangle =(-\frac{3}{64{\pi }^{2}{\varepsilon }_{0}^{2}{R}^{6}}\alpha (A;0)\alpha (B;0)\hat{E})/(-\frac{11\hslash c{\kappa }^{11/2}{\kappa ^{\prime} }^{1/2}}{960{\pi }^{4}{\varepsilon }_{0}^{2}R}\alpha (D;\kappa )\alpha (A;\kappa ^{\prime} ))$$

6 $${\rm{\Delta }}ENZ/\langle {\rm{\Delta }}ENZ\rangle =\frac{3\times 960{\pi }^{2}}{64\times 11\hslash c{\kappa }^{11/2}{\kappa ^{\prime} }^{1/2}{R}^{5}}\alpha (A;0)\alpha (B;0)\hat{E}/(\alpha (D;\kappa )\alpha (A;\kappa ^{\prime} ))$$

We plug in vacuum permittivity ε_0_ = 8.85 × 10^−12^ F·m^−1^, reduced Planck constant *ħ* = 1.055 × 10^−34^ J·s = 6.58 × 10^−16^ ev·s, speed of light transmission in vacuum *c* = 3 × 10^8^ m/s. The above equation can be simplified as:

7 $$\begin{array}{ccc}{\rm{\Delta }}ENZ/\langle {\rm{\Delta }}ENZ\rangle  & = & \frac{3\times 960{\pi }^{2}}{64\times 11\hslash c{\kappa }^{11/2}{\kappa ^{\prime} }^{1/2}{R}^{5}}\alpha (A;0)\alpha (B;0)\hat{E}/(\alpha (D;\kappa )\alpha (A;\kappa ^{\prime} ))\\  & = & 177.64\times \alpha (A;0)\alpha (B;0)\hat{E}/(\alpha (D;\kappa )\alpha (A;\kappa ^{\prime} )ev)\end{array}$$

We assume the lowest-energy transition E^ε^(ε = A, B) as 2.8 ev for both donor and acceptor molecules as the energy corresponding to wavelength at 443 nm, which is close to emission of PFluAnt and absorption of PFOBT. The scaled energy Ê is thus 2.8 ev. As a following,

8 $${\rm{\Delta }}ENZ/\langle {\rm{\Delta }}ENZ\rangle =497.4\times \alpha (A;0)\alpha (B;0)/(\alpha (D;\kappa )\alpha (A;\kappa ^{\prime} ))$$

The calculation suggest that the relative scale of van der Waals interaction and RET-induced intermolecular pairing force depends on the relative magnitudes of static isotropic electric dipole polarizabilities and dynamic polarizabilities at resonant frequency for both donor and acceptor chromophores. Because there is no precise value of α(A;0)/α(A;*κ*) where *κ* stands for wave vector at resonant frequency, we use the value of ~1/20 from relevant refs 27 and 28 as the ratio for both donor and acceptor as a rough estimation. Their values were plugged in above equation,

9 $${\rm{\Delta }}ENZ/ < {\rm{\Delta }}ENZ > =\frac{497.4}{400}=1.24$$

## Electronic supplementary material

supporting information
