# Supplementary material for: Observation of aggregation triggered by Resonance Energy Transfer (RET) induced intermolecular pairing force
Source: Sci Rep. 2017 Jul 20;7:5970. doi: 10.1038/s41598-017-05157-8 (PMC5519756; doi:10.1038/s41598-017-05157-8)
Supplement: Supplementary file 1 — supporting information [file 41598_2017_5157_MOESM1_ESM.doc]

**Observation of aggregation triggered by Resonance Energy Transfer (RET) induced intermolecular pairing force**

Xiaoyong Pan^1*^, Weizhi Wang^2^, Lin Ke^1^, Nan Zhang^1^

^1^2 Fusionopolis Way, Innovis, #08-03, Institute of Materials Research and Engineering (IMRE), Singapore 138634

^2^State Key Laboratory of Molecular Engineering of Polymers, Collaborative Innovation Center of Polymers and Polymer Composite Materials, Department of Macromolecular Science, Fudan University, Shanghai, China, 200433

To whom correspondence should be addressed: Xiaoyong Pan ([panx@imre.astar.edu.sg](mailto:panx@imre.astar.edu.sg)).

**a b**


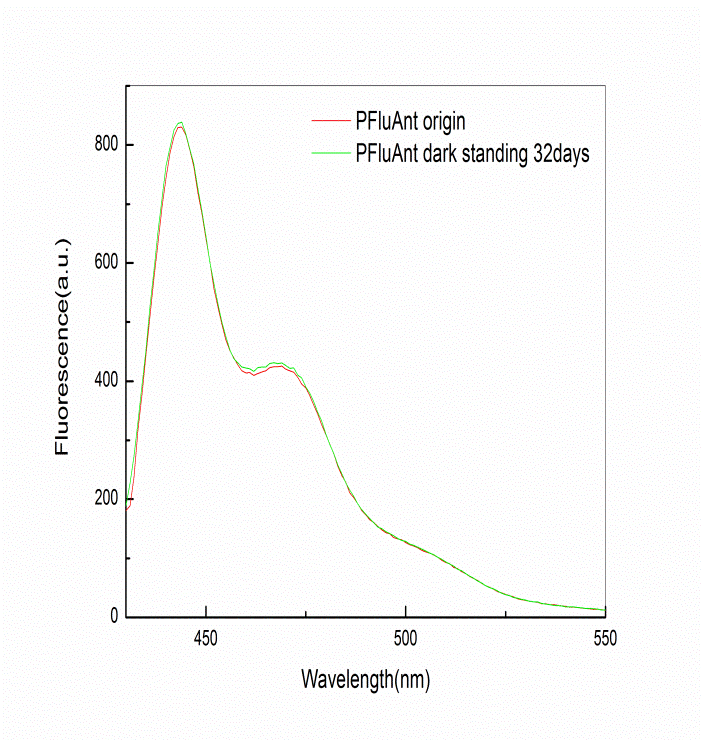

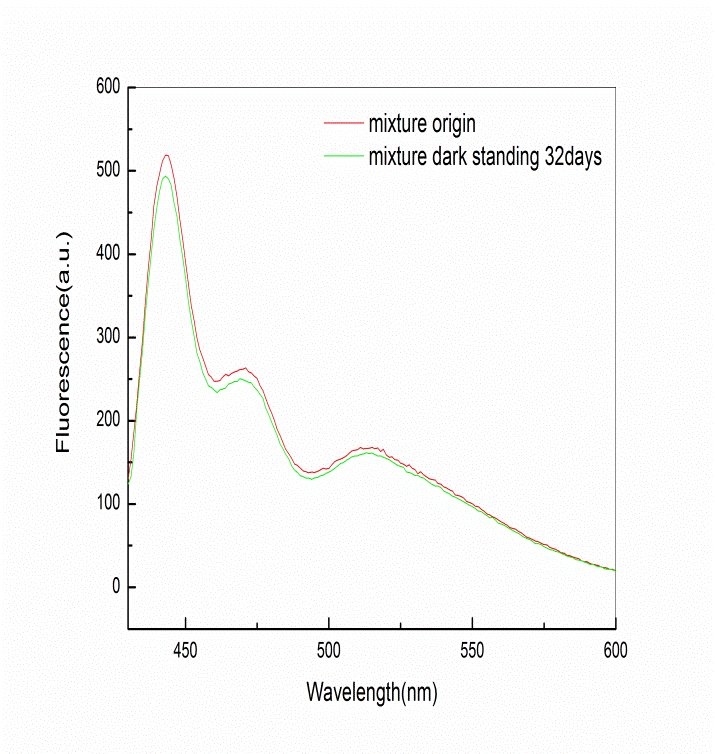


**c**


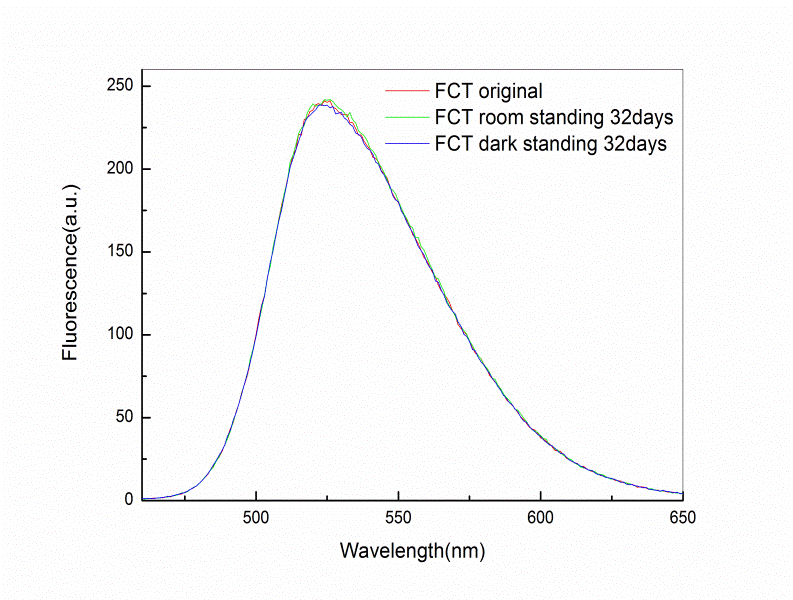


**Figure S1.**  **Fluorescence properties of PFluAnt solution, PFOBT solution or PFluAnt&PFOBT mixture.** **a,** Fluorescence spectra of PFluAnt solution standing in dark area after 32 days’ standing time. **b,** Fluorescence spectra of PFluAnt&PFOBT mixture standing in dark area after 32 days’ standing time. **c,** Fluorescence spectra of PFOBT under room illumination as well as standing in dark area. (More spectra have been collected while only two were shown for the sake of clearance.)

**a b**


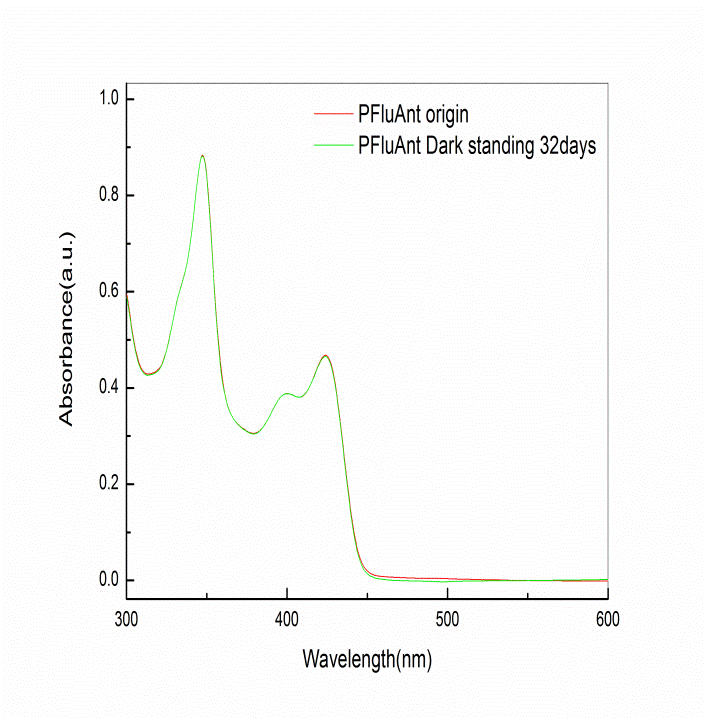

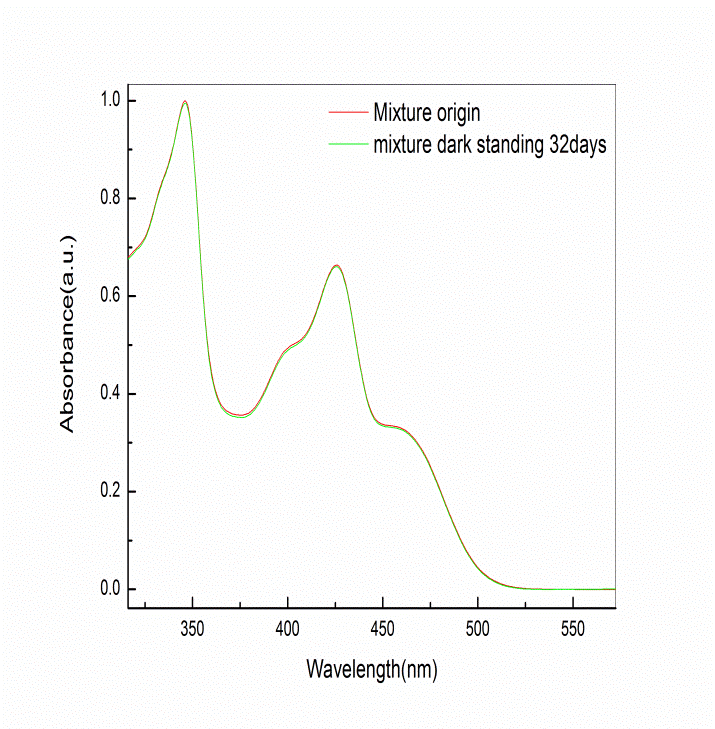


**Figure S2.** **UV-Vis absorption properties of PFluAnt solution or PFluAnt&PFOBT mixture. a,** UV-Vis absoption spectra of PFluAnt solution standing in dark area for 32 days. **b,** UV-Vis absoption spectra of PFluAnt&PFOBT mixture standing in dark area after 32 days.

**a**


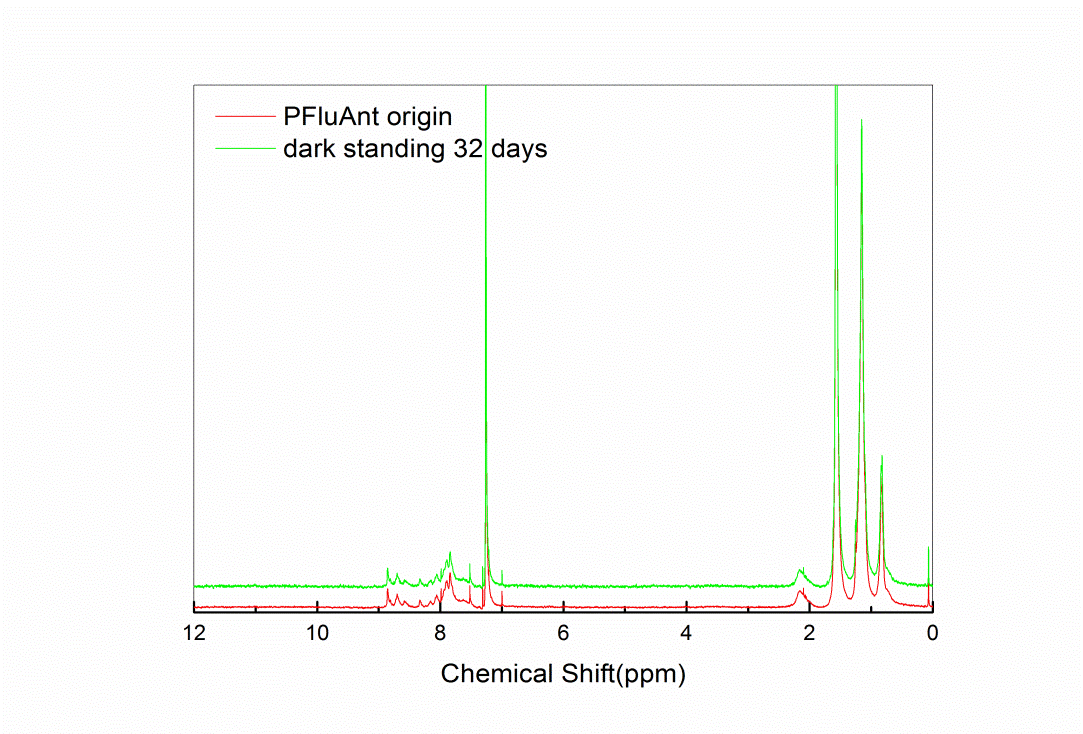


**b**


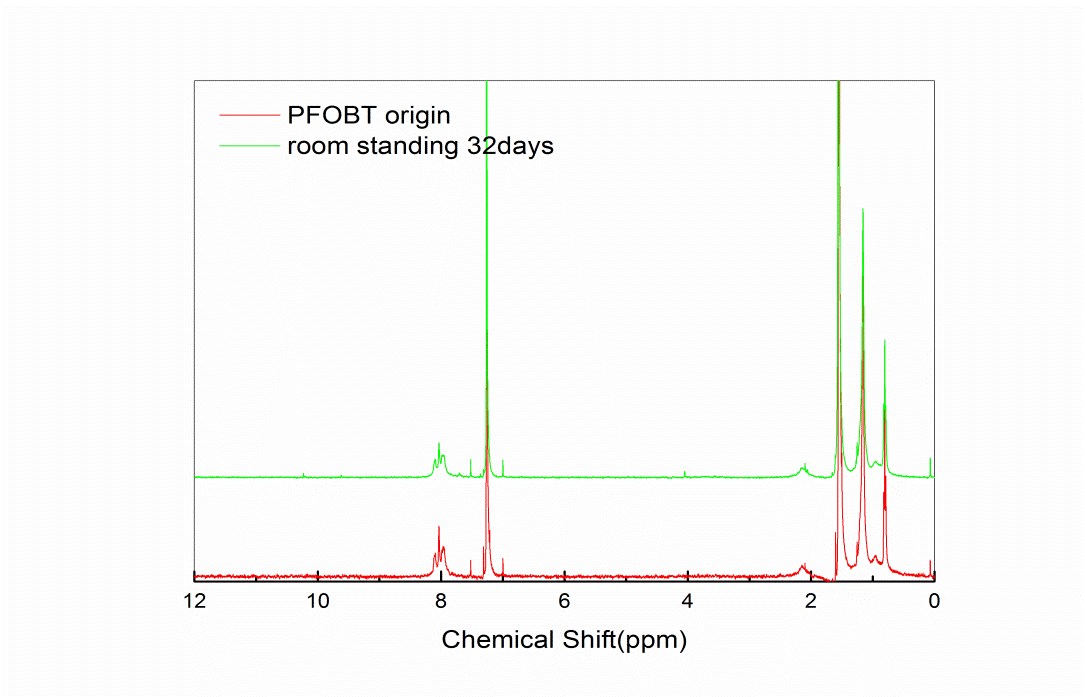


**Figure S3.** **NMR spectra of PFluAnt of PFOBT solution. a,** NMR spectra of original PFluAnt in CDCl3 and that after 32 days’ dark standing. **b,** NMR spectra of original PFOBT in CDCl3 and that after 32 days’ room illumination.

**a b**


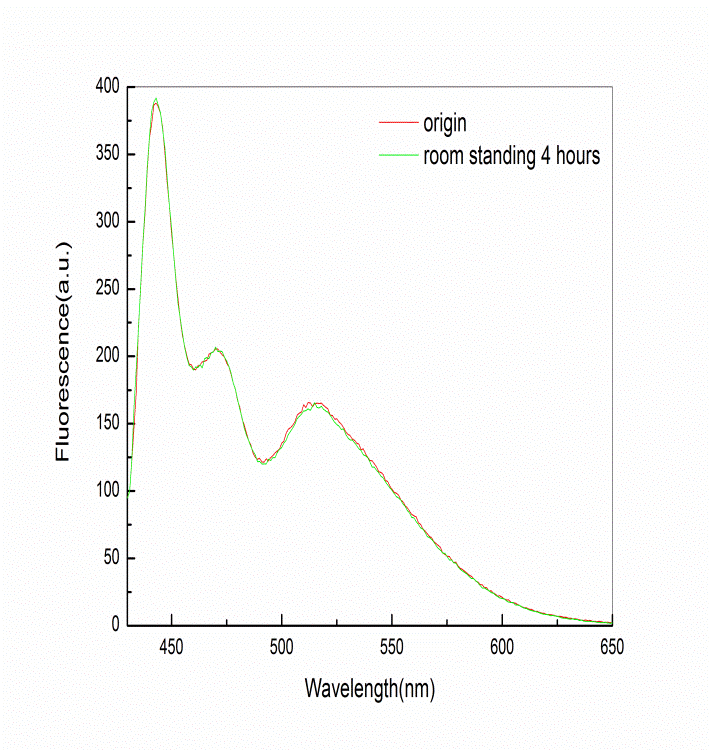

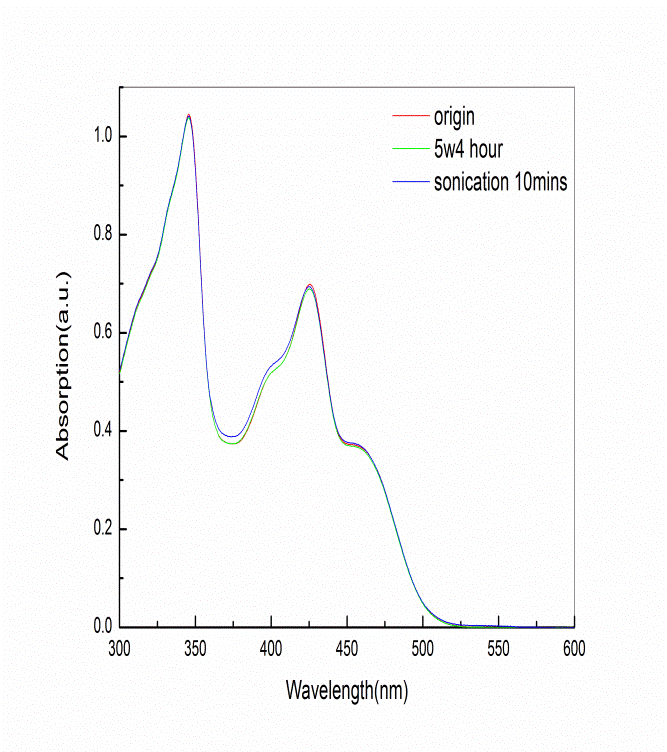


**Figure S4.** **Fluorescence and UV-Vis absorption spectra of PFluAnt&PFOBT mixture.** **a,** Fluorescence spectra of PFluAnt&PFOBT mixture after 4 hours’ standing under room illumination. **b,** UV-Vis absorption spectra of PFluAnt&PFOBT mixture after irradiance with 5 W desk lamp for 4 hours.
